# Laparoscopic Vesicovaginal Fistula Repair Using Kumar’s Knotless Technique: Short-Term Experience From a Tertiary Care Center

**DOI:** 10.7759/cureus.82083

**Published:** 2025-04-11

**Authors:** Lalit Kumar, Surendra Kumar, Sakshi Agarwal, Anuja Thakur, Sameer Trivedi

**Affiliations:** 1 Department of Urology, Institute of Medical Sciences, Banaras Hindu University, Varanasi, IND; 2 Department of Obstetrics and Gynecology, Institute of Medical Sciences, Banaras Hindu University, Varanasi, IND

**Keywords:** knotless, laparoscopic, urogenital fistula, vesicovaginal fistula (vvf), v-loc barbed suture

## Abstract

Introduction: The laparoscopic (lap) technique is well-established for the repair of vesicovaginal fistula (VVF). However, its main limitation is a lack of expertise in intracorporeal suturing. This retrospective study presents a lap VVF repair using Kumar’s knotless technique with V-Loc™ (Medtronic, MN, USA) barbed suture and compares its results with the open VVF repair technique.

Methods: In this retrospective study, we included 14 consecutive patients receiving VVF repair operated on by open conventional repair (open group) or lap knotless techniques (lap group) between March 2020 and March 2024. The data were recorded retrospectively.

Results: Comparative analysis between the lap (n = 8) and open (n = 6) groups demonstrated that lap repair significantly reduced intraoperative blood loss (48.75 ± 15.00 mL vs. 108.33 ± 28.71 mL, respectively, p< 0.001), drain duration (3.13 ± 0.35 days vs. 7.17 ± 0.41 days, respectively, p< 0.001), postoperative pain (3.13 ± 1.55 vs. 6.17 ± 1.47, respectively, p < 0.01), and hospital stay (3.38 ± 0.74 days vs. 7.83 ± 0.98 days, respectively, p< 0.001). No significant differences were found between the lap and open groups in terms of mean age (33.25 ± 5.76 vs. 37.33 ± 5.77, respectively, p = 0.103), fistula size (1.56 ± 0.80 cm vs. 1.58 ± 0.37 cm, respectively, p = 0.938), or timing of bowel function return (15.25 ± 2.33 vs. 16.83 ± 3.71, respectively, p = 0.193). These results suggest that lap repair improves recovery outcomes without compromising clinical efficacy.

Conclusion: Lap VVF repair using Kumar’s knotless technique with V-Loc™ barbed suture represents a feasible and safe approach to managing VVF. The technique offers a viable alternative to traditional methods, particularly in settings with limited experience with lap intracorporeal suturing and access to robotic surgery.

## Introduction

Vesicovaginal fistula (VVF) poses a multifaceted challenge to patients, spanning physical, psychological, and socioeconomic dimensions. In developed nations, VVF is predominantly linked to gynecologic surgeries and stands as the most prevalent form of urogenital fistula. These complications typically arise following iatrogenic injuries during specific procedures, such as hysterectomy or lower-segment cesarean section [1]. However, VVF can also occur due to obstructed labor, uterine rupture, following radiotherapy treatment and other radical procedures for pelvic tumors or pelvic surgeries, or following trauma to the urinary bladder (usually iatrogenic) [2].

The global incidence of VVF ranges between 0.3% and 2% [3]. Addressing VVF often necessitates surgical intervention, with two primary approaches being through the abdomen and vagina for fistulas above the trigonal area and for low-lying fistulas, respectively [4]. Regardless of the chosen surgical technique, fundamental repair principles include meticulous bladder-vagina separation, water-tight, tension-free fistula closure in two to three layers, between which the autologous tissues like omental, epiploic, and peritoneal tissues for abdominal techniques while labial fibrofatty pad (Martius flap), peritoneum, and gracilis muscle are usually placed for vaginal approach. Some studies have used synthetic materials like fibrin glue, gelatin matrix, cyanoacrylates, collagen, cellulose, and small intestinal submucosa as interposition flaps. Still, they pose the risk of immunogenicity as compared to autologous flaps. These flaps are usually indicated for recurrent and complex fistulas at the expense of prolonging operative time and donor site morbidity. It is essential to have continuous urine drainage through postoperative catheterization [5]. Although the abdominal technique offers greater versatility, it carries inherent risks, such as bleeding and long incisions leading to increased pain, more prominent surgical scars, and delayed convalescence and recovery. Conversely, the transvaginal technique mitigates risks associated with the transabdominal approach and is typically suited for fistulae that are low-lying and do not require concurrent ureteric reimplantation. Understanding each approach’s nuances and related complications is crucial to providing optimal care for individuals suffering from VVF [4,6].

Over the last few decades, there has been tremendous improvement in minimally invasive surgeries for VVF, particularly in addressing complex cases that cannot be easily managed through traditional transvaginal approaches. The groundbreaking work of Nezhat et al. in 1994 marked the inception of this transformative surgical method when they performed the first laparoscopic (lap) repair of VVF [7]. Subsequent studies have built upon this foundation, introducing modifications and innovations to enhance surgical outcomes. For example, Von Theobald et al. pioneered the omental J flap technique [8], and Miklos et al. demonstrated the applicability of laparoscopy in recurrent VVF cases [9]. Furthermore, technological advances have led to the adoption of robot-aided minimally invasive surgical procedures, which have shown excellent results [10].

Despite the effectiveness of robotic surgery in managing VVF, its limited availability, familiarity, and higher costs in India and other developing countries have led urologists to rely on the open transabdominal and lap approaches predominantly. In this retrospective study, we aimed to assess the efficacy of a modified lap approach for VVF repair. We analyzed various perioperative factors, including patient age, etiology, fistula location and size, the time interval between the causative event and surgery, operative duration, blood loss, and postoperative recovery measures. We focused on reducing the time of intracorporeal suturing and facilitating effective suturing through a knotless technique using barbed sutures (V-Loc™, Medtronic, MN, USA). Our goal was to highlight the outcomes of this simplified approach, offering a practical alternative for VVF repair in settings where robotic surgery is not readily accessible.

## Materials and methods

From March 2020 to March 2024, a comprehensive approach was implemented at the Institute of Medical Sciences, Banaras Hindu University, Varanasi, to address VVF repair involving 14 consecutive patients retrospectively. A single surgeon performed all surgeries. These patients were separated into two groups according to surgical approach: eight in the lap group and six in the open surgery (open) group. Ethical approval was obtained from the Institute of Medical Sciences, Banaras Hindu University, Varanasi, IMS/IEC/2024/7313, and all patients provided informed written consent in their preferred language.

Preoperative evaluations included cystoscopy to assess fistula characteristics and vaginoscopy to examine vaginal condition. Inclusion criteria included patients undergoing VVF repair by the lap or open approach. Comprehensive laboratory and imaging tests were performed to ensure patient eligibility for surgery, including complete blood count, kidney and liver function tests, urine routine microscopy and culture, computed tomography urography, and cysto-vaginoscopy. Preoperative data regarding fistula characteristics, type of surgery, operative time, suturing time, drain and hospital duration, and complications were recorded and entered into a Microsoft Excel (Microsoft Corporation, Redmond, Washington, United States) spreadsheet using discharge papers and patient admission files. Statistical analysis was conducted using independent samples t-tests and chi-square tests, with p-values of <0.05 considered statistically significant.

Technical steps

The surgical procedure for lap VVF repair involved a multi-step process designed to minimize patient trauma while ensuring a successful surgical outcome. The initial preparation stage began with patient intubation and positioning in the lithotomy position to facilitate cystoscopy and bilateral ureteric catheterization of different colors with 5-Fr ureteric catheters. A distinctively colored catheter was placed into the fistula to ensure easy identification during the procedure. A Foley catheter was inserted to monitor urine output during surgery. The ureteric catheters were placed bilaterally and fixed with a Foley catheter. To ensure proper sterilization, the vaginal cavity was packed with povidone-iodine-soaked cotton gauze.

In the surgical setup phase, the patient was repositioned to the supine position with both upper limbs placed by their sides. The pneumoperitoneum was created, and a 12-mm supraumbilical port was inserted for a camera. Two additional ports, one 12 mm and one 5 mm, were placed 8-10 cm laterally and slightly inferiorly to facilitate the lap procedure. An additional 5-mm port was sometimes inserted in the iliac fossa to facilitate surgical dissection and suturing. The surgical team adjusted the patient into a low-lying lithotomy position with a steep Trendelenburg tilt to enhance access to the pelvic area as the bowel fell.

The abdominal cavity was first inspected to assess the location of the fistula. The peritoneum was opened in the area between the bladder and the vagina near the fistula site with the help of a tug on the Foley catheter and feeling a lifted vaginal swab on a sponge holder instrument. Dissection around the fistula followed, with careful separation of the urinary bladder from the vagina to create sufficient space for the repair. A small posterior cystotomy was made near the fistula site to expose the fistulous opening, with the colored ureteric catheter aiding in identification. The dissection continued to create space between the posterior wall of the urinary bladder and the anterior vaginal wall. The vaginal opening was then closed using continuous intracorporeal suturing with 2-0 V-Loc™ barbed sutures and a knotless technique. The bladder was sutured similarly with a knotless technique using continuously running 3-0 V-Loc™ barbed sutures. An interposition of omental flap or appendices epiploic was placed and fixed with 3-0 V-Loc™ sutures at the interlunation of suture lines of the vagina and bladder to reinforce the repair further. Under gravity, 200-300 mL of normal saline was instilled gently into the bladder to ensure a secure and waterproof closure. A drain was placed in the pelvis after thoroughly suctioning the collected fluid. The created pneumoperitoneum was terminated, and the ports were closed. Finally, the vaginal packing was replaced with fresh povidone-iodine-soaked cotton gauze.

Postoperative care included the administration of intravenous antibiotics and diclofenac for pain management. Patients could start liquids once bowel sounds returned, typically on the first postoperative day, and advance to semisolid and solid foods by the second day. The discharge occurred between three and five days for patients in the lap VVF group after drain output became minimal and the drain was removed.

Open surgery was performed using a standard transvesical approach. These patients were usually discharged seven to nine days after surgery, after removing bilateral infant feeding tubes and the drain. A per urethral catheter was generally removed at two to three weeks post-surgery. Patients were advised to refrain from sexual intercourse for three months to allow adequate healing of the repair site.

## Results

In the lap group, supratrigonal fistulas were the most prevalent type, varying in size from 0.5 to 3 cm. Etiology ranged from total abdominal hysterectomy (TAH) to obstructed labor, and time intervals from etiology to surgery spanned three to nine months. Surgical outcomes indicated operative duration varied between 85 and 130 minutes and intraoperative blood loss from 30 to 75 mL. According to the visual analog scale (VAS), postoperative pain ranged from one to six. Most patients had a drain duration of three days, with one patient experiencing a bladder spasm, leading to an extended hospital stay and increased postoperative pain (Tables 1, 2).

**Table 1:** Demographic and clinical characteristics of patients in the lap VVF group TAH: total abdominal hysterectomy; LSCS: lower segment caesarean section; VVF: vesicovaginal fistula; lap: laparoscopic

S. No.	Age (years)	Etiology	Site of fistula	Size of fistula (cm)	Time interval between etiology and surgery (months)
1	42	TAH	Supratrigonal	1	5
2	38	TAH	Supratrigonal	1	4.5
3	25	LSCS	Supratrigonal	3	3.5
4	32	Cesarean hysterectomy	Vesicocervical	2	6
5	39	Lap hysterectomy	Supratrigonal	1	3
6	30	Obstructed labour	Supratrigonal	2	9
7	30	Cesarean section	Supratrigonal	2	6
8	30	TAH	Supratrigonal	0.5	5

**Table 2:** Perioperative surgical outcomes in the lap VVF group POD: postoperative day; VAS: visual analog scale; TOV: trial of void; VVF: vesicovaginal fistula; lap: laparoscopic

S. No.	Operative duration (min)	Blood loss (mL)	Drain (mL, POD 1)	Drain duration (days)	Complications	Clavien Dindo grade	Postoperative pain (VAS)	Hospital stay (days)	Timing of TOV (days)
1	120	50	25	3	Nil	1	3	3	14
2	90	40	40	3	Nil	1	2	3	14
3	125	75	50	4	Bladder spasm	2	6	5	21
4	110	60	35	3	Nil	1	4	3	14
5	100	40	25	3	Nil	1	2	3	14
6	130	50	40	3	Nil	1	5	3	14
7	95	45	50	3	Nil	1	2	3	14
8	85	30	25	3	Nil	1	1	3	14

In the open VVF group, operative duration ranged from 105 to 116 min, and intraoperative blood loss ranged from 75 to 150 mL, with the 150-mL cases necessitating a blood transfusion. Notably, complications were recorded for three patients: one had a bladder spasm, one required a blood transfusion, and one developed postoperative ileus. According to recovery metrics, postoperative VAS pain scores varied between four and eight, and hospital stays ranged from seven to nine days. Drain duration was consistently seven days, with a trial of void (TOV) at 14 days for most, extending to 21 days for those with postoperative ileus and bladder spasms (Tables 3, 4).

**Table 3:** Demographic and clinical characteristics of the patients in the open VVF group TAH: total abdominal hysterectomy; VVF: vesicovaginal fistula

S. No.	Age (years)	Etiology	Site of fistula	Size of fistula (cm)	Time interval between etiology and surgery (months)
1	32	TAH	Supratrigonal	1.5	4
2	36	TAH	Supratrigonal	2	6
3	41	Transvaginal hysterectomy	Infratrigonal	2	3.5
4	31	Cesarean section	Supratrigonal	2	9
5	45	TAH	Supratrigonal	1	5.5
6	39	TAH	Supratrigonal	1.5	7.5

**Table 4:** Perioperative surgical outcomes in the open VVF group POD: postoperative day; VAS: visual analog scale; TOV: trial of void; VVF: vesicovaginal fistula

S. No.	Operative time (min)	Blood loss (mL)	Drain (mL, POD 1)	Drain duration (days)	Complications	Clavien Dindo grade	Postoperative pain (VAS)	Hospital stay (days)	Timing of TOV (days)
1	107	100	75	7	Nil	1	6	7	14
2	110	150	110	8	Blood transfusion	2	8	9	14
3	105	90	50	7	Nil	1	5	7	14
4	116	125	60	7	Ileus	2	7	8	21
5	109	75	40	7	Nil	1	4	7	14
6	112	110	100	7	Bladder spasm	2	7	8	21

Comparative analysis between lap and open vesicovaginal fistula groups

Comparative analysis showed no significant difference in mean age between the lap and open groups (33.25 ± 5.76 years vs. 37.33 ± 5.77 years, respectively, p = 0.103). Similarly, no significant differences were found when comparing fistula site (predominantly supra-trigonal in both groups (87.5% vs. 83.3%, p = 0.654)), fistula size (1.56 ± 0.80 cm vs. 1.58 ± 0.37 cm, p = 0.938), and the time interval from etiology to surgery (5.38 ± 1.94 months vs. 5.92 ± 2.12 months, p = 0.564) between the lap and open groups, respectively. However, intraoperative blood loss was significantly lower in the lap group (48.75 ± 15.00 mL vs. 108.33 ± 28.71 mL, respectively, p< 0.001), and drain duration was shorter (3.13 ± 0.35 days vs. 7.17 ± 0.41 days, respectively, p< 0.001) compared to the open group. Furthermore, in the lap group, postoperative pain was significantly lower than in the open group (3.13 ± 1.55 vs. 6.17 ± 1.47, respectively, p< 0.01), and patients had a shorter hospital stay (3.38 ± 0.74 days vs. 7.83 ± 0.98 days, respectively, p< 0.001). Trail of void (TOV) return was similar between the lap and open groups (15.25 ± 2.33 days vs. 16.83 ± 3.71 days, respectively, p = 0.193). These results indicate that lap surgery offers significant advantages in reduced blood loss, shorter recovery time, lower postoperative pain, and quicker hospital discharge. In addition, the groups showed similar results in other clinical parameters and outcomes, such as age, fistula size, and timing of TOV. The lap and open VVF groups experienced success rates of 100%, with no recurrence among the study sample (Table 5).

**Table 5:** Comparative analysis of baseline characteristics and perioperative parameters SE: standard error; IQR: interquartile range; VAS: visual analog scale; TOV: trial of void; lap: laparoscopic

Clinical variable	Lap group	Open group	p-value
Age (years)	33.25 ± 5.76, SE: 2.04, IQR: 7.00	37.33 ± 5.77, SE: 2.36, IQR: 8.25	0.103
Site of fistula	Supratrigonal (87.5%)	Supratrigonal (83.3%)	0.654
	Vesico-cervical (12.5%)	Infra trigonal (16.7%)	
Size of fistula (cm)	1.56 ± 0.80, SE: 0.28, IQR: 1.50	1.58 ± 0.37, SE: 0.15, IQR: 0.75	0.938
Time interval between etiology and surgery (months)	5.38 ± 1.94, SE: 0.69, IQR: 3.00	5.92 ± 2.12, SE: 0.87, IQR: 3.00	0.564
Operative duration (min)	107.88 ± 15.80, SE: 5.59, IQR: 25.00	109.83 ± 3.71, SE: 1.52, IQR: 7.00	0.681
Blood loss (mL)	48.75 ± 15.00, SE: 5.30, IQR: 20.00	108.33 ± 28.71, SE: 11.72, IQR: 45.00	< 0.001
Drain duration (days)	3.13 ± 0.35, SE: 0.12, IQR: 0.00	7.17 ± 0.41, SE: 0.17, IQR: 0.00	< 0.001
Postoperative pain (VAS)	3.13 ± 1.55, SE: 0.55, IQR: 2.00	6.17 ± 1.47, SE: 0.60, IQR: 2.00	< 0.01
Hospital stay (days)	3.38 ± 0.74, SE: 0.26, IQR: 0.00	7.83 ± 0.98, SE: 0.40, IQR: 1.00	< 0.001
Timing of TOV (days)	15.25 ± 2.33, SE: 0.82, IQR: 7.00	16.83 ± 3.71, SE: 1.52, IQR: 7.00	0.193

## Discussion

Our study highlights the successful application of the knotless lap repair technique for VVF. The method utilizes three trocars, usually alongside a limited posterior cystotomy, with the help of monopolar electrocautery and V-Loc™ barbed suture [11]. The “limited cystostomy” or “Mini-O’Conor vesicostomy, as elaborated by Rizvi et al. [12], offers a minimally invasive alternative to the classical O’Conor approach [13], which includes a great cystotomy extending down to the posterior bladder wall. Although the O’Conor technique provides good visualization, it complicates subsequent lap suturing, prolongs operative duration, and may contribute to postoperative bladder spasms. Conversely, the total “extravesical approach,” as elaborated by Miklos and Moore [14], avoids cystotomy altogether; however, it compromises the visualization of ureteric orifices, increasing the traumatic risk during bladder closure, particularly near the orifices. The limited cystotomy approach balances these considerations by providing adequate visualization while decreasing the chances of complications associated with the standard O’Conor cystostomy. In addition, such cases experience neither hematuria nor bladder spasms requiring anticholinergics.

In lap surgeries, intracorporeal suturing poses significant challenges, particularly in the narrow confines of the pelvic region. Conventional sutures may cause tissue necrosis and reduced wound strength due to excessive stretching during knot-tying and increased operative time. Barbed sutures, which eliminate the requirement of knot-tying, have gained popularity because of their perceived advantages in providing a secure closure and reducing operative time [15]. Previously used in robotic VVF repair, the efficacy of barbed sutures in lap settings is promising. This report provides a potentially valuable solution to the complexities of lap suturing using V-LocTM barbed sutures [16].

This study highlights the substantial benefits of the lap knotless suture technique with V-Loc™ suture for VVF repair. In the lap group, consisting primarily of patients with supratrigonal fistulas, the mean operative time was 107.88 min, the mean intraoperative blood loss was 48.75 mL, and the average length of hospital stay was 3.38. In contrast, the open group had a mean operative time of 109.83 min, a mean blood loss of 108.33 mL, and a mean hospital stay of 7.83 days. Therefore, the lap group exhibited better outcomes than the open group regarding blood loss and length of hospital stay, whereas operative duration was similar in both groups. Furthermore, the lap group experienced significantly less postoperative pain (VAS scores of 3.13 vs. 6.17) and shorter drain durations (3.13 days vs. 7.17 days, respectively) than the open group.

The use of V-Loc™ suture, which eliminates the need for knot-tying, keeps tension all along the suture line without loosening the previous bite of the suture. Watertight closure with minimal drain output might play a crucial role in the above-mentioned improvements by reducing tissue irritation and inflammation, thus contributing to quicker recovery. We also had limited cystostomy during VVF dissection, as reported by Giannakopoulos et al. [17].

Compared with other lap VVF studies using conventional suture techniques, the advantages of the V-Loc™ knotless suture technique become even more apparent. Shah et al. reported a mean operative time of 145 min and a mean blood loss of 180-200 mL with conventional sutures, achieving an 86% success rate [16]. Similarly, Mahapatra et al. noted a mean operative time of 166 min and a mean blood loss of 125 mL, with a 91.7% success rate [18]. A meta-analysis of lap VVF repair by Ghosh et al. reported similar results [19]. In contrast, our study achieved a 100% success rate and mean intraoperative blood loss of <50 mL with V-Loc™ suture, highlighting its efficacy in VVF repair. Reducing operative time, blood loss, and postoperative complications associated with V-Loc™ suture makes it an alternative, superior choice for lap VVF repair, enhancing surgical efficiency and patient outcomes [12]. These findings underscore the importance of adopting advanced suturing techniques to improve surgical success and patient recovery. However, V-Loc™ suture may lead to higher costs, with limited availability.

Compared with the robotic VVF repair, the strengths and limitations of each approach become more evident. Robotic-assisted lap VVF repair offers enhanced precision and accuracy, fewer hand tremors, improved dexterity, and better three-dimensional visualization, leading to high success rates and minimal postoperative complications. For example, a study by Sotelo et al. reported excellent outcomes with robotic VVF repair, demonstrating a high success rate and minimal morbidity [13]. However, the steep learning curve, higher initial and maintenance costs, and limited availability of robotic systems, particularly in developing countries, are significant disadvantages. In contrast, our study’s knotless surgical technique with a lap approach, while also minimally invasive, offers a more accessible and cost-effective alternative with comparable success rates. The use of V-Loc™ suture with the knotless technique further simplifies the procedure, making it an attractive, safe, feasible, and efficacious option for surgeons and patients [2,3]. In their recent systematic review and meta-analysis, Hafermann et al. reported these advantages of V-Loc™ barbed sutures in gynecologic surgeries. However, literature regarding the use of the knotless technique in lap VVF repair is currently unavailable. One reason for this research gap may be surgeon apprehension regarding the urinary tract, as it is a dynamic organ that remains filled with urine, making it susceptible to urine leaks, unlike the vaginal vault [20].

The strength of our study lies in its modified lap VVF repair surgical technique. In addition, all surgeries were performed by a single surgeon, which enhances reliability and reduces heterogeneity. We also compared Kumar’s knotless technique with open VVF repair. The V-Loc™ knotless suture technique with limited cystostomy in the lap group offers valuable insights into improving surgical outcomes.

Certain limitations of the present study, such as its retrospective nature and small sample size, limit the generalizability of our findings. The relatively short follow-up period may also not capture long-term outcomes. Additionally, the single-surgeon approach may not reflect broader surgical practices, necessitating further prospective randomized research with larger multicenter cohorts and extended follow-up durations to validate these findings. Other limitations include a lack of cost-effective analysis and comparison with standard lap VVF repair.

## Conclusions

Our results suggest that lap VVF repair using Kumar’s knotless technique with V-Loc™ barbed sutures is a feasible and safe approach to managing VVF. This technique offers a viable alternative to traditional methods, such as open and lap VVF repair, without hampering the success rate or increasing complication rates. This modified technique lowers the hospital stay and complications in the immediate postoperative period. This technique is particularly suitable for surgeons with limited experience with lap intracorporeal suturing methods and access to robotic surgery.
